# Four-Component Statistical Copolymers by RAFT Polymerization

**DOI:** 10.3390/polym16101321

**Published:** 2024-05-08

**Authors:** Dimitrios Vagenas, Stergios Pispas

**Affiliations:** Theoretical and Physical Chemistry Institute, National Hellenic Research Foundation, 48 Vassileos Constantinou Avenue, 11635 Athens, Greece; dimitrisv98@gmail.com

**Keywords:** statistical copolymers, multicomponent polymers, bio-inspired polymers, RAFT polymerization, responsive copolymers, polyelectrolytes, amphiphilic copolymers, proteins

## Abstract

This manuscript serves as the starting point for in-depth research of multicomponent, statistical, methacrylate-based copolymers that potentially mimic the behavior of proteins in aqueous solutions. These synthetic macromolecules are composed of specially chosen comonomers: methacrylic acid (MAA), oligoethylene glycol methyl ether methacrylate (OEGMA_475_), 2-(dimethylamino)ethyl methacrylate (DMAEMA) and benzyl methacrylate (BzMA). Monomer choice was based on factors such as the chemical nature of pendant functional groups, the polyelectrolyte/polyampholyte and amphiphilic character and the overall hydrophobic–hydrophilic balance (HLB) of the obtained quaterpolymers. Their synthesis was achieved via a one-pot reversible addition fragmentation chain transfer (RAFT) polymerization in two distinct compositions and molecular architectures, linear and hyperbranched, respectively, in order to explore the effects of macromolecular topology. The resulting statistical quaterpolymers were characterized via ^1^H-NMR and ATR-FTIR spectroscopies. Their behavior in aqueous solutions was studied by dynamic (DLS) and electrophoretic light scattering (ELS) and fluorescence spectroscopy (FS), producing vital information concerning their self-assembly and the structure of the formed aggregates. The physicochemical studies were extended by tuning parameters such as the solution pH and ionic strength. Finally, the quaterpolymer behavior in FBS/PBS solutions was investigated to test their colloid stability and biocompatibility in an in vivo-mimicking, biological fluid environment.

## 1. Introduction

Macromolecules are a general class of compounds that is strongly related to both synthetic and biological chemistry, two research fields that exhibit great scientific interest in the 21st century due to several factors. Such compounds are polymers and proteins, respectively, displaying certain particularities concerning their synthesis, isolation and properties on a physicochemical and biological level. On the one hand, proteins can be considered as biopolymers, composed of repeating building blocks, the amino acids, and they participate actively in numerous biological processes of living organisms. Proteins are widely considered to be the building blocks of life, as they display impeccable functionality, specificity and versatility due to their unique property of folding and adopting a three-dimensional structure. This is determined to some extent by the sequence and type of amino acids along the protein chain; the amino acids comprise the proteins’ primary structure. On the other hand, polymers are either synthetic or natural macromolecular compounds that display fascinating, tailored properties in aqueous solutions, which are mainly derived from their self-assembly, creating intriguing nanostructures depending on the chemical nature of their components, their composition and the existing inter- or intramolecular interactions. Polymer science is gradually focusing on research that involves nanoparticle formation and nanostructure characterization and defining the polymers’ role in a variety of biomedical applications. This trend can be considered anything but accidental. It is due to the emerging development of innovative methods for polymer synthesis, which contribute to the well-defined structure and properties of the polymers produced, also adding considerable ease to the synthesis itself.

In particular, a plethora of studies consider controlled radical polymerization methods, such as RAFT (reversible addition fragmentation chain transfer polymerization), through which control is accomplished regarding the mode of introduction of monomeric units with side groups of different polarities along the chain. In this way, it becomes possible to synthesize copolymers of various macromolecular architectures/topologies, with different densities of monomeric units (linear, star-shaped, hyperbranched), through which well-defined, self-organized nanostructures are subsequently achieved in a solution state [1]. Statistical (or random) copolymers are a class of synthetic macromolecules that are characterized by the random distribution of the different monomeric units along the polymer chain. Their synthesis can be achieved via a one-step reaction by applying the RAFT technique, making it possible to copolymerize two or more monomers simultaneously. Even though the particular polymeric class does not provide well-defined nano-assemblies, intriguing supramolecular interactions take place when self-assembly is induced, causing the formation of nanodomains with distinct morphologies. Random copolymers tend to self-organize in a way that mimics the solution behavior of proteins, raising significant scientific interest into the investigation of their properties’ similarities. In fact, upward trends are also observed in studies concerning the encapsulation of proteins in such nanostructures or their co-assembly with polymer chains, showing promising results in biomedicine- and biotechnology-related applications [2,3].

The striking property of most proteins to induce their folding brings synthetic, statistical copolymers to the forefront, as results from recent studies demonstrate that they can have a biological effect, functioning synergistically with proteins, contributing to their stabilization in non-polar environments and even forming proton transport channels in biological membranes [4,5,6]. Undoubtedly, the sequence of structural units along the polymer chain should affect the overall polymer–protein interactions. Nevertheless, some studies point out the fact that some properties such as folding will not be due to the sequence, but to more general characteristics such as the composition of the structural units, heterogeneity and concentration in solution [7]. Indeed, some findings demonstrate the direct effect of homopolymers when complexed with proteins during folding. In particular, interactions between characteristic groups in the side chains and amino acids contribute to the “unfolding” (stretching) of polypeptide chains [8]. Moreover, it is highlighted that there is slow development in terms of successful protein stabilization in vitro, which should serve as a springboard for long-term study of the use of synthetic polymers in this direction.

According to recent research, copolymers that would potentially perform the aforementioned roles during their interaction with proteins are considered to be those of methacrylate esters, in particular those of amphiphilic character, i.e., with hydrophilic and hydrophobic segments/domains [9]. Amphiphilic copolymers are considered a recent case study in the field of polymer science and synthetic chemistry. Due to the incompatibility of hydrophilic and hydrophobic domains, self-assembly is induced through the formation of nanostructures in aqueous solutions, which are composed of a type of intramolecular or intermolecular organization with a hydrophobic core and a hydrophilic shell [10,11]. The molecular architecture of amphiphilic copolymers has an active role both in self-assembly in aqueous solutions and in the functions they can perform subsequently in the form of nanoparticles. Few reports consider linear and hyperbranched amphiphilic copolymers both in terms of their synthesis and biological applications, e.g., transport of bioactive substances such as proteins and peptides [12]. In essence, multicomponent copolymers with the aforementioned architectures and statistical distribution of their structural units (segments) exhibit folding behavior in aqueous solutions, which resembles that of proteins lacking a well-defined tertiary structure, also known as “Intrinsically Disordered Proteins” (IDPs) [13,14].

In this work, the synthesis of linear, statistical copolymers and their hyperbranched analogues is attempted. They consist of four different methacrylate monomer components, where each one serves a certain purpose depending on their characteristic side chains. We use the term “statistical” since it denotes by definition the sequential distribution of two or more different monomers along polymer chains, where their incorporation obeys known statistical laws (although, in our case, the exact monomer distribution is not known), and it is broader than the term “random”, because the latter is used when the monomer sequential distribution follows Bernoullian statistics. In most cases, the terms “statistical” and “random” are used in the literature interchangeably without a definition of the kind of statistics the sequence of monomers in the polymer chain obeys. This is extremely difficult to determine in most cases even for linear two-component copolymers, let alone for four-component quaterpolymers or copolymers with non-linear/branched architectures. Oligo(ethylene glycol) methacrylate (OEGMA_475_) serves the role of the hydrophilic component in relation to hydrophilic non-ionic amino acids. Taking into account its ability to stabilize macromolecular systems in aqueous media and its apparent biocompatibility, its presence along polymeric chains raises great interest [15,16,17]. Methacrylic acid (MAA) was chosen due to its acidic and hydrophilic nature, and specific reference should be made to its ability to respond to pH alterations, an ability that 2-(dimethylamino)ethyl methacrylate (DMAEMA) segments also exhibit. On the one hand, MAA segments become more hydrophobic at low pH values, whereas the exact opposite phenomenon occurs for DMAEMA segments. Such responsive systems may have certain applications related to studies of endosomal membrane disruption, targeting the protection of biotherapeutics and evolving drug delivery in general [18,19]. Together, these segments give a polyampholyte character to the copolymer chain that is reminiscent of similar protein characteristics. The fourth monomer that is incorporated in the present copolymers is benzyl methacrylate (BzMA), which contains a phenyl group and adds hydrophobic character to the final quaternary copolymers. Physicochemical studies of the resulting quaterpolymers are undertaken in order to collect preliminary information concerning their behavior in water and simulated biological environments. 

## 2. Materials and Methods

### 2.1. Materials

Monomers, methacrylic acid (MAA), 2-(dimethylamino)ethyl methacrylate (DMAEMA), oligo(ethylene glycol) methacrylate (OEGMA) (average M_n_ = 475 g/mol), benzyl methacrylate (BzMA) and cross-linking agent ethylene glycol dimethacrylate (EGDMA, difunctional monomer) were purified by passing them through a column packed with inhibitor-removing resins 311,340 and 311,332 from Sigma Aldrich (Athens, Greece). 2,2-azobis(isobutyronitrile) (AIBN) was recrystallized from methanol and used as the radical initiator. 1,4-dioxane (99.8% pure) was chosen as the solvent of the reaction and dried using molecular sieves. 4-cyano-4-(phenylcarbonothioylthio) pentanoic acid (CPAD), i.e., the chain transfer agent of the reaction, pyrene and deuterated dimethyl sulfoxide-d_6_ (dDMSO) were used as received (all from Sigma-Aldrich, Athens, Greece). Dialysis tubing membranes (MEMBRA-CELL) from regenerated cellulose of MWCO 3500 with a diameter of 22 mm were obtained from SERVA (Heidelberg, Germany).

### 2.2. Synthesis of Linear and Hyperbranched Statistical Quaterpolymers

The synthesis procedure was planned in such a way as to fabricate statistical quaterpolymers of two different monomer compositions in two discrete macromolecular architectures, linear and hyperbranched, and this was achieved via one-pot RAFT polymerization. Purified monomers, AIBN and CPAD were placed in a 25 mL round-bottom flask and were all dissolved in 1,4-dioxane. The CPAD:AIBN molar ratio was adjusted to 2:1, and the targeted polymer molecular weight was 20,000 g/mol for the linear copolymers. After homogenization of the mixture under stirring, the flask was sealed with a rubber septum, and deoxygenation of the polymerization solution was achieved via nitrogen bubbling for 20 min. Then, the flask was put in an oil bath at 70 °C under stirring and left there for 24 h for the monomers to polymerize. Afterwards, the flask was kept directly at −20 °C for 20 min and finally exposed to air, terminating the polymerization. Impurities such as oligomers or unreacted monomers were removed through dialysis against distilled water for 2 days, using dialysis membranes with pores of molecular weight cut-off of 3500 Dalton. The final step of the purification of copolymers was the water removal and their forthcoming drying, which was achieved via the utilization of a rotary evaporator. The experimental procedure described above was executed as is for the synthesis of hyperbranched quaterpolymers, with the only difference being the addition of the crosslinker ethylene glycol dimethacrylate (EGDMA) in the flask before the initiation of the reaction. Ιts amount was calculated from the EGDMA:CPAD molar ratio, which was regulated at 1.2:1. It has to be noted that EGDMA was also purified using the same inhibitor-removing resins as those applied to the other four monomers.

### 2.3. Self-Assembly of Quaterpolymers in Aqueous Media

All four quaterpolymers were diluted directly in deionized water in order to investigate their ability to self-assemble and their overall physicochemical behavior in aqueous solutions. For each copolymer, a stock solution was prepared with a constant polymer concentration chosen at 0.5 × 10^−4^ g/mL and pH = 7 and left overnight in order to be equilibrated. This experimental study was extended by aiming to investigate the potential influence of solution pH in the assembly of the quaterpolymers; aqueous solutions of pH 3 and 10 were thus prepared for all copolymers by adding the appropriate amount of HCl 0.1 M and NaOH 0.1 M, respectively. All solutions were filtered through hydrophilic PVDF 0.45 μm disposable filters before light-scattering measurements.

### 2.4. FBS Interactions with Quaterpolymers

Quaterpolymers were mixed with fetal bovine serum (FBS) to study any possible interactions with blood proteins. In brief, 150 μL of aqueous solutions of the quaterpolymers were added to 1.5 mL of FBS:PBS solution with 1:9 *v*/*v* ratio (10% *v*/*v* FBS–90% *v*/*v* PBS). The samples were measured by dynamic light scattering (DLS) 1 h and 24 h after their preparation, and the collected data were compared with those of neat FBS. All samples were filtered using 0.45 μm porosity hydrophilic PVDF filters.

### 2.5. Characterization Methods

#### 2.5.1. Proton Nuclear Magnetic Resonance Spectroscopy (^1^H-NMR)

^1^H NMR measurements of quaterpolymer solutions were performed on a Varian 300 (300 MHz) spectrometer using Vjnmr rev. 3.2A software for spectra acquisition. Deuterated dimethyl sulfoxide-d_6_ was used as the solvent for sample preparation (c ≈ 10 mg/mL). Chemical shifts are given in parts per million (ppm) using tetramethylsilane as the internal reference, and the results were analyzed with MestReNova software v. 6.0.2 from MestReLabs (Santiago de Compostela, Spain).

#### 2.5.2. Attenuated Total Reflectance-Fourier Transform Infrared (ATR-FTIR) Spectroscopy

ATR-FTIR spectra of dry solid quaterpolymer samples were recorded on a Bruker (Billerica, MA, USA) Equinox 55 Fourier transform spectrometer, equipped with a single-bounce ATR diamond accessory (Dura-Samp1IR II by SensIR Technologies, Danbury, CT, USA). Each spectrum was received as the average of 64 scans collected in the 5000 to 500 cm^−1^ spectral range and at 4 cm^−1^ resolution.

#### 2.5.3. Dynamic Light Scattering (DLS)

DLS studies were carried out using an ALV/GS-3 compact goniometer system (ALV GmbH, Hessen, Germany) with a JDS Uniphase 22 mW He–Ne laser, operating at 632.8 nm wavelength. The system is equipped with an ALV/LSE-5003 light-scattering electronics unit used for stepper motor drive and limit switch control and an ALV-5000/EPP multi-τ correlator including 288 channels. The obtained autocorrelation functions (and the simultaneously recorded light-scattering intensity) were the average of five measurements at a goniometer angle of 90°, analyzed by the cumulants method and the CONTIN algorithm. All aqueous solutions were filtered through 0.45 µm hydrophilic PVDF filters before measurements.

#### 2.5.4. Electrophoretic Light Scattering (ELS)

Zeta potential values, which are directly related to the surface charge of polymer particles in solution, were measured by electrophoretic light-scattering experiments conducted on a Nano Zeta Sizer instrument from Malvern, which is equipped with a 4 mW He–Ne, operating at 633 nm and a scattering angle of 173°. Each measurement was an average of 20 repeated scans, and the obtained data were analyzed by the Smoluchowski equation.

#### 2.5.5. Fluorescence Spectroscopy (FS)

Fluorescence measurements were conducted using a NanoLog Fluorimeter (Horiba Jobin Yvon, Kyoto, Japan), with a laser diode as the excitation source (NanoLED, 440 nm, pulse width 100 ps) and a UV TBX-PMT series detector (250–850 nm) by Horiba Jobin Yvon. Critical aggregation concentration was investigated through this characterization technique. Sample preparation for quaterpolymer solutions provided for the successive dilution of a stock solution to finally obtain eleven copolymer solutions in the concentration range of 5 × 10^−4^ to 5 × 10^−9^ g/mL. The stock solution preparation protocol was retained the same as described above. The appropriate pH was adjusted where necessary for all eleven solutions, and then pyrene solution in acetone, at a ratio of 1 µL/mL, was added to each vial. The samples were kept at rest for 24 h to ensure encapsulation of pyrene into the hydrophobic domains of the polymer aggregates and evaporation of acetone. The excitation wavelength used for the measurements was 335 nm. Emission spectra were recorded in the spectral range of 355–640 nm. The ratio I_1_/I_3_, i.e., the ratio of intensities of the first and third vibronic peaks in pyrene fluorescence spectra, was utilized to access the hydrophobicity of the pyrene environment in the polymer solutions.

## 3. Results

### 3.1. Synthesis and Qualititative Characterization of Quaterpolymers

**Scheme 1:**
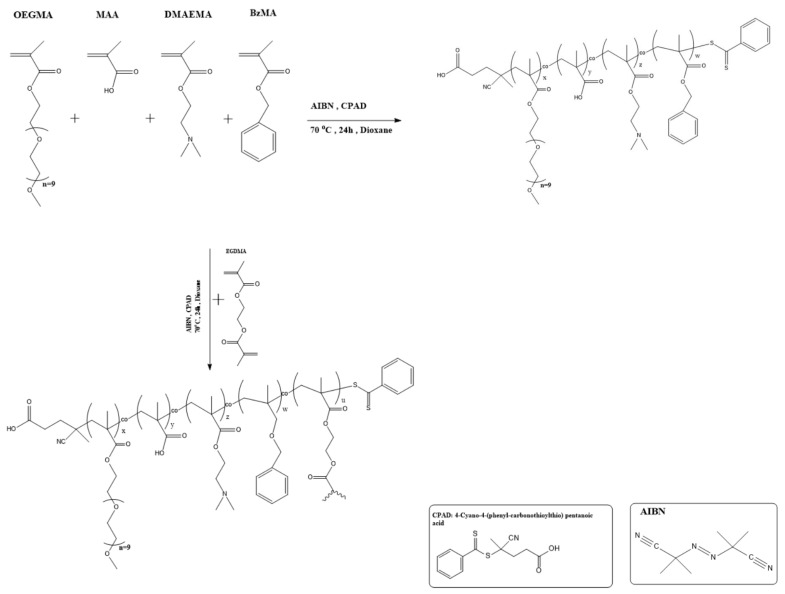
Synthesis route for obtaining linear (LT1, LT2) and hyperbranched (HT1, HT2) statistical quaterpolymers via one-pot RAFT polymerization.

The first step of the studies was the synthesis of four statistical quaterpolymers, and RAFT was chosen as the polymerization method, as shown in Scheme 1. The concept is based on the synthesis of copolymers of two different monomer compositions, each corresponding to two macromolecular architectures, linear and hyperbranched. The quaterpolymers are of the type P(MAA-co-DMAEMA-co-OEGMA-co-BzMA) and H-P(MAA-co-DMAEMA-co-OEGMA-co-BzMA), respectively. The two distinct stoichiometric wt% compositions [MAA:DMAEMA:OEGMA:BzMA] of the quaterpolymers are [25:25:40:10] and [20:20:40:20]. The design of the aforementioned compositions was delivered in accordance with the desirable polyelectrolyte and amphiphilic character of the resulting quaterpolymers, and their molecular weight was targeted as 20,000 g/mol, a value that usually corresponds to the molecular weight of several common proteins. CPAD was chosen to be the chain transfer agent, as its utility to the polymerization of a broad spectrum of methacrylates is proven [20]. In the following sections, quaterpolymers will be reported with their abbreviated form: LT1 / HT1 is assigned to the [25:25:40:10] composition and LT2 / HT2 to the [20:20:40:20] composition, where the capital letter “L” stands for linear, and the letter “H” stands for hyperbranched quaterpolymer architecture.

**Figure 1:**
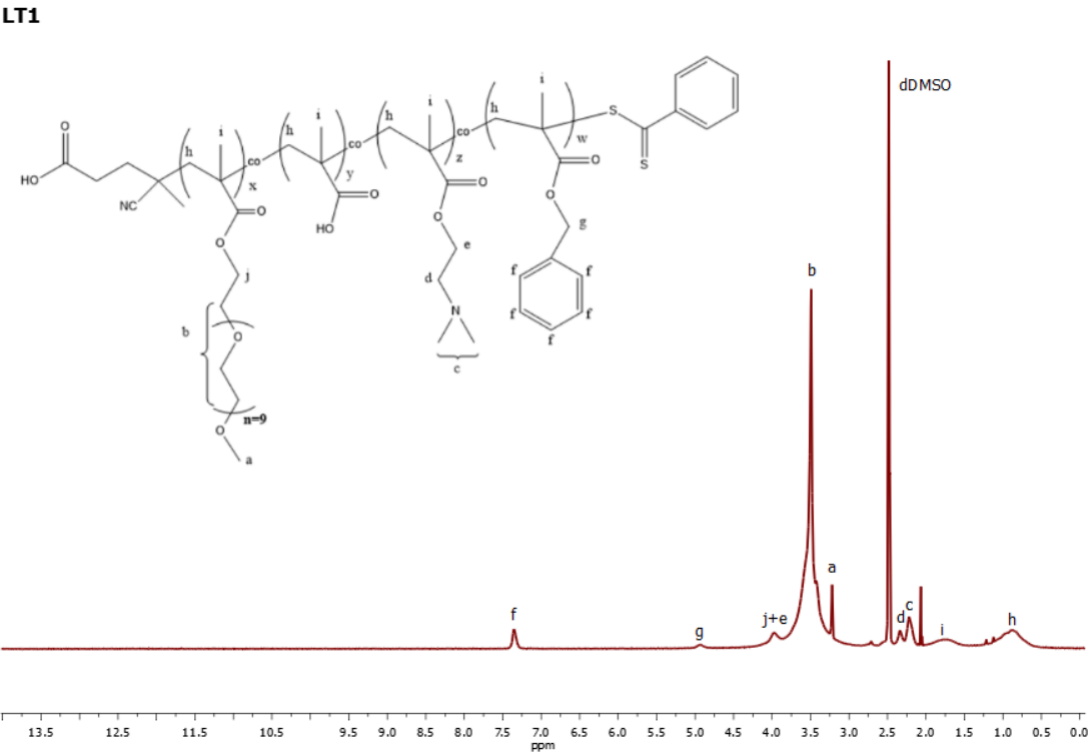

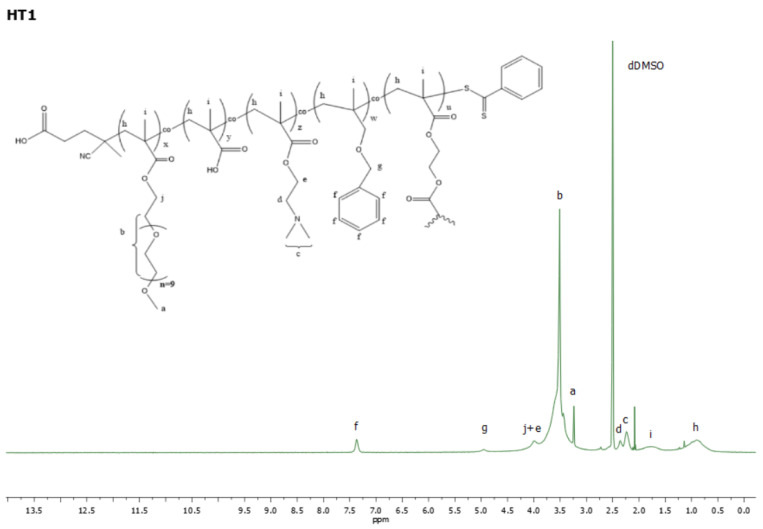
^1^H-NMR spectra of LT1 (red, (**top**)) and HT1 (green, (**bottom**)).

Molecular characterization of the obtained quaterpolymers was attempted via ^1^H-NMR and ATR-FTIR measurements. Size-exclusion chromatography (SEC) could not be applied on such copolymers due to the presence of methacrylic acid, which demands specialized equipment with specific columns and mobile phase not available in our lab. The resulting quaterpolymers were glassy solids with an amphiphilic and polyampholyte nature that could not be dissolved in any organic solvent but only dimethyl sulfoxide (DMSO). Thus, its deuterated analogue was utilized to obtain polymer solutions for ^1^H-NMR experiments. During the ^1^H-NMR spectra analysis of the four quaterpolymers, a peak around 12 ppm, which is characteristic of carboxylic groups, was not observed, probably because of the special conformation/aggregation state of the quaterpolymers (due to the strong polar nature of the solvent hydrophobic aggregation as well complexation between the acidic and basic segments of the quaterpolymers that may take place). Thus, it was considered necessary to employ the ATR-FTIR technique for the molecular characterization of the quaterpolymers in the solid state, in order to ensure the presence or absence of methacrylic acid within the quaterpolymer chains. Such limitations prevented the detailed molecular characterization of the four statistical quaterpolymers; therefore, a qualitative analysis was the only option to ensure the presence of all monomers and extract some information concerning the result of the polymerizations in each case. LT1 is a representative quaterpolymer that is utilized to showcase the qualitative characterization results obtained in this part of our study, as all four copolymers had similar composition characteristics, and their spectra are presented in the Supplementary Materials. The ^1^H-NMR spectrum of LT1 is presented in Figure 1, where all the characteristic peaks are assigned to specific protons. The peak at 7.36 ppm is characteristic for protons of an aromatic ring and proves the presence of benzyl methacrylate segments. The peaks at 3.49 and 3.22 correspond to -OCH_2_CH_2_- and -OCH_3_ group protons, respectively, proving the presence of OEGMA segments, and finally, the peaks at 2.22 and 2.33 ppm correspond to -N(CH_3_)_2_ and -(C=O)-OCH_2_- groups, respectively. The repeated methylene groups of the polymer backbone and the attached methyl groups are also depicted as relatively broad peaks at 0.88 and 1.74 ppm. The absence of peaks at 4.5–6.5 ppm, except for the one at 4.94, which is attributed to the protons of -OCH_2_- group, proves the absence of vinyl groups and the success of the dialysis procedure.

**Figure 2:**
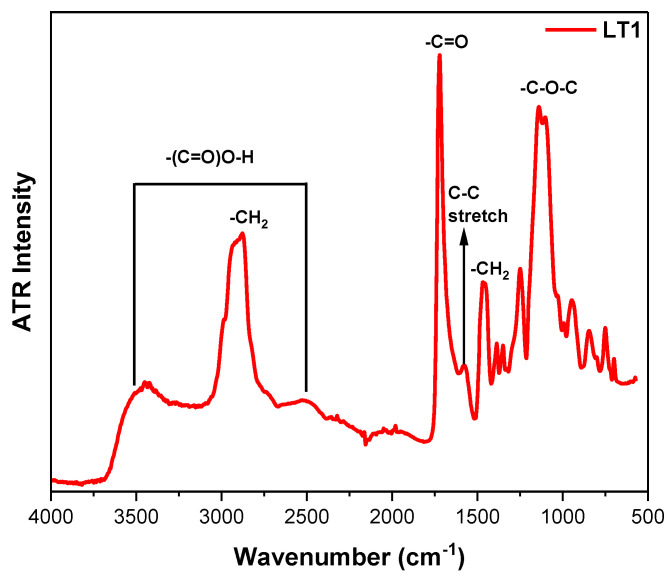
ATR−FTIR spectrum of LT1 quaterpolymer.

ATR-FTIR analysis was performed in order to mainly detect carboxylic groups confirming the presence of methacrylic acid units in the quaterpolymers. In Figure 2, the characteristic broad band at 2500–3500 cm^−1^ is assigned to stretching vibrations of carboxylic acid hydroxyls, qualitatively confirming the presence of methacrylic acid units. 

### 3.2. Physicochemical Characterization of the Quaterpolymer Assembly in Aqueous Media

#### 3.2.1. Critical Aggregation Concentration (CAC) Determination

**Figure 3:**
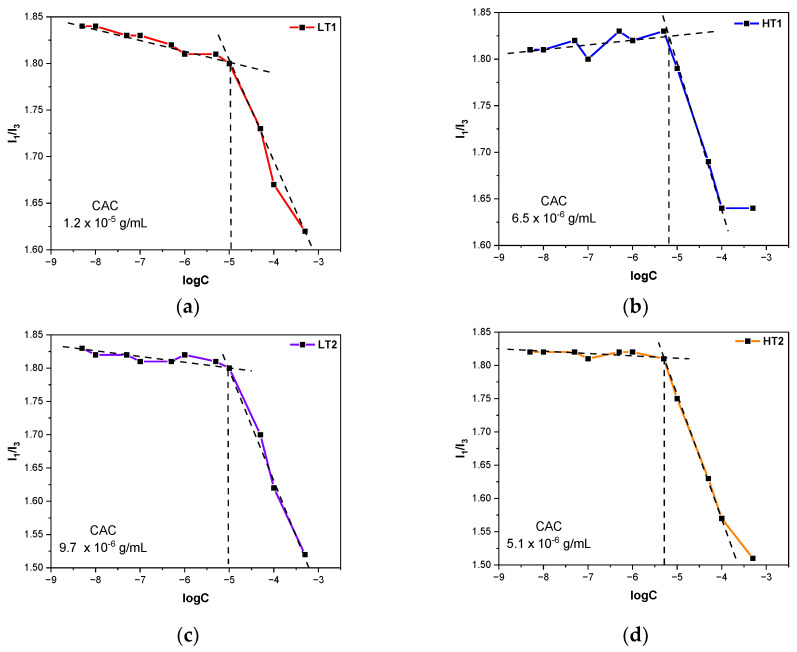
CAC determination for LT1 (**a**), HT1 (**b**), LT2 (**c**) and HT2 (**d**).

The self-assembly of the statistical methacrylate quaterpolymers in aqueous solutions is studied by following carefully designed protocols of characterization methods in an exact order. Fluorescence measurements were the starting point of this series of experiments, aiming to gain information concerning the amphiphilicity of the copolymers and to investigate the possible aggregation of copolymer chains at a particular critical concentration (CAC), an effect that is exhibited in many cases by the majority of methacrylate amphiphilic copolymers. The execution of such measurements requires the utilization of a fluorescent probe such as pyrene, which exhibits a characteristic emission spectrum depending on the polarity of its surroundings. Pyrene is a hydrophobic, organic compound that tends to be entrapped into hydrophobic domains of a polymer assembly. The intensities of the first (371 nm) and third peak (382 nm) of the pyrene emission spectrum shift according to the polarity of the environment in which pyrene is accommodated, and therefore, the ratio (I_1_/I_3_) contains polarity-relevant information. Low values of the ratio indicate a hydrophobic micro-environment, whereas high values indicate a hydrophilic one. The critical aggregation concentration (CAC) is determined by calculating the I_1_/I_3_ ratio for eleven different concentrations and then plotting the data in function with polymer concentration. The intersection between the two tangent lines of the data curves, where a sharp decrease in the ratio is usually observed, is the point denoting the critical aggregation concentration of the corresponding copolymer [21]. Figure 3 presents the plotted data concerning the I_1_/I_3_ ratio as a function of the logarithm of the quaterpolymer concentration and the corresponding CAC values at the initial pH (pH~7).

As presented in Figure 3, all four quaterpolymers exhibit relatively hydrophilic character in their corresponding solution state at neutral pH, a reasonable outcome taking into account the low hydrophobic content of both series, 10% for the first and 20% for the second one, respectively. To be more specific, the I_1_/I_3_ ratio values of the highest tetrapolymer concentration (5 × 10^−4^ g/mL) investigated range from 1.52 to 1.64, which can be attributed to the formation of aggregates with small hydrophobic character (probably with small hydrophobic domains), due to the high OEGMA and low BzMA content in all copolymer samples. CAC values are relatively low, indicating that the formation of aggregates is initiated at states of high dilution in neutral pH, a finding that can be considered as a primary indication of their potential stability [22]. Moreover, a differentiation at CAC values between linear and hyperbranched copolymers is observed and ranges approximately at one order of magnitude. Therefore, the aggregation phenomenon is triggered at a higher dilution for hyperbranched copolymers, a finding consistent with the fact that copolymer chains coexist at a shorter area, resulting in more compact aggregates. Such measurements were held also for copolymer solutions at acidic and basic pH values (3 and 10), and the results are discussed later in Section 3.3.

Since I_1_/I_3_ tends to change significantly from low to high concentrations, it can be preliminarily assumed that both series of quaterpolymers self-assemble in aqueous media due to hydrophobic interactions and adapt certain conformations within the aggregates.

#### 3.2.2. Structural Studies on Quaterpolymer Self-Assembly at Neutral pH

**Figure 4:**
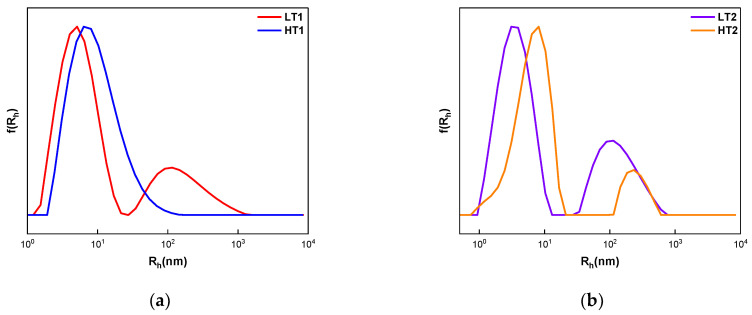
Size distributions from CONTIN analysis of DLS correlation functions for quaterpolymer LT1/HT1 (**a**) and LT2/HT2 (**b**) aqueous solutions at 25 °C and pH = 7.

Having ensured that the four quaterpolymers self-assemble via the formation of aggregates in aqueous media, their physicochemical and structural characterization was followed, aiming to gain more information on the structure and behavior of these aggregates. Aqueous solutions of the four tetrapolymers were prepared via their direct dissolution in deionized water, as described in the experimental section, with the concentration regulated at 0.5 mg/mL while the solution pH remained neutral (pH~7). Dynamic light-scattering (DLS) measurements were conducted, determining parameters such as the apparent hydrodynamic radius (R_h_), the scattered light intensity, the size polydispersity index (PDI) and the corresponding size distributions in the aqueous solutions. The intensity-weighted size distributions in the solutions of the four quaterpolymers are presented at pairs of linear and hyperbranched architectures in Figure 4 based on copolymer composition.

Size distributions from CONTIN analysis of DLS correlation functions provide important information concerning the spontaneous self-organization of copolymers in aqueous solutions. First and foremost, the molecular architecture factor is reflected in both pairs of quaterpolymers. Hyperbranched copolymers may not have the same molecular weight as their linear analogues. The crosslinker functions as a monomeric unit and connects polymeric chains; thus, the actual molecular weight is higher. Such an assumption is reflected in both groups of plots in Figure 4, as the curves for hyperbranched copolymers are shifted to larger R_h_ values compared to linear ones, and their corresponding scattered light intensities (I_90_°) are approximately double (×1.7) (Table 1), indicating nanoparticles of greater mass in these cases. Moreover, for the quaterpolymer pair LT1/HT1, where the hydrophobic content is the minimum, a strong differentiation between nanoparticle species formed in solution is observed. The size distribution of LT1 depicts the existence of both small-sized (probably single chains or dimer/trimer aggregates) and large-sized (multichain aggregates) structures, whereas HT1 presents a broad monomodal distribution of sizes, a result that is in accordance with the branched copolymer architecture. Linear, amphiphilic copolymers tend to self-organize in a way that non-polar units aggregate together to avoid contact with water molecules, resulting in the formation of a hydrophobic domain. The polar units disperse around it, forming an overall micelle-type arrangement within the aggregates [23]. When it comes to hyperbranched copolymers, their nanoparticles should adopt a more globular and compact structure than their corresponding linear analogues, since the segment density is already higher than that of linear chains [24,25]. Their size distribution, and specifically the nanoparticle species and populations, depend on factors such as the content of hydrophobic segments incorporated in a polymeric chain and the branching degree. It must be stated that in this study, the synthesized hyperbranched quaterpolymers have a relatively small branching degree (due to the relatively low EGDM/CTA ratio utilized). This is chosen because we should consider a light stereochemical hindrance between aggregating segments and polymeric chains, favoring their folding as it happens in proteins with their specific molecular conformations. For the second quaterpolymer pair LT2/HT2, the influence of the increased hydrophobic content is observed, especially in the case of the hyperbranched copolymer, as a second population of nanoparticles with larger dimensions appears. The doubling of BzMA hydrophobic segments’ content from HT1 to HT2, in combination with the hyperbranched architecture, provokes the formation of nanoaggregates in the second case. The mobility of the polymeric chains decreases near branching points, exposing the hydrophobic segments to a greater extent. This in turn induces increased hyperbranched quaterpolymer aggregation due to the much-favored hydrophobic interactions and results in nanoaggregates with a large hydrodynamic radius. In addition, the increase in the non-polar content is depicted readily in the case of the linear copolymers, as the presence of large aggregates becomes more pronounced.

### 3.3. Influence of Solution pH on Quaterpolymer Assembly

**Figure 5:**
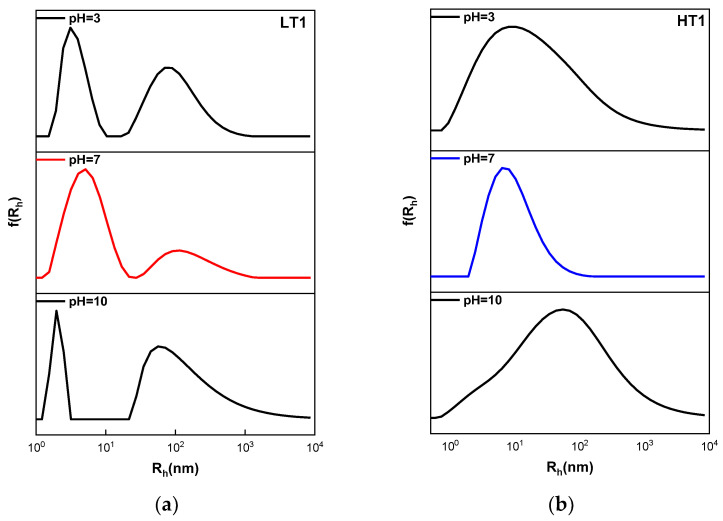

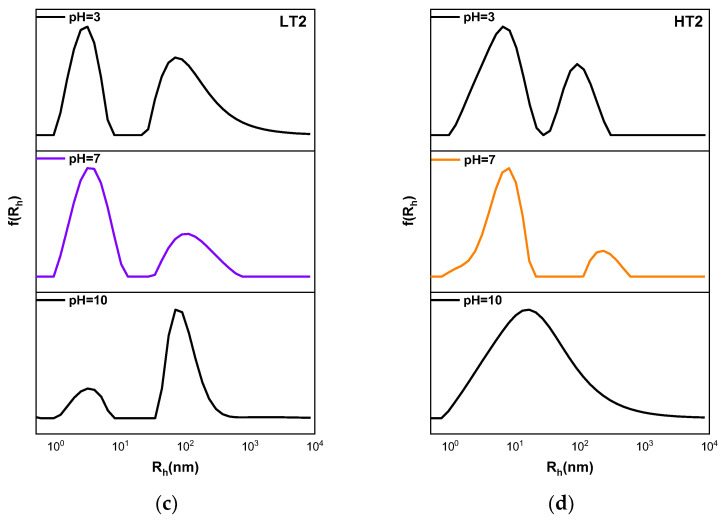
Size distributions from CONTIN analysis of DLS data for quaterpolymers LT1 (**a**), HT1 (**b**), LT2 (**c**) and HT2 (**d**) at three different pH values, at 25 °C.

The synthesized quaterpolymers contain (in each pair) a similar amount of DMAEMA and MAA units, monomers that have been utilized in several studies for their pH-responsive character. Therefore, the role of solution pH on the self-assembly of the quaterpolymers was investigated next. DMAEMA units carry a tertiary amino group, which is partially protonated at neutral pH (pK_a_~7.6), and impart a hydrophilic character to the copolymers that are incorporated. At acidic pH values (pH~3), the amino groups are fully protonated, intensifying hydrophilicity of the polymer, whereas at basic conditions (pH~10), deprotonation occurs, inducing hydrophobic characteristics, which favors aggregation between polymer chains [26]. On the other hand, methacrylic acid (MAA) is an anionic monomer in neutral pH, with a pK_a_ value of around 4.8, as it contains a pendant carboxylic group. At basic pH, ionization of the pendant carboxylic groups occurs, causing the swelling of the respective aggregates due to electrostatic repulsion between negatively charged groups. On the contrary, when acidic conditions are established in solution, the collapsed state of polymeric chains dominates [27]. The co-existence of these two monomers in the same polymeric chain imparts ampholytic character to the quaterpolymers, therefore provoking electrostatic interactions and complexation between oppositely charged groups depending on solution pH. In order to study the overall influence of pH on the assembly behavior of quaterpolymers, dynamic and electrophoretic light-scattering measurements were conducted. The results are summarized in Table 1, and the respective size distributions for each copolymer in three different pHs are presented in Figure 5.

The analysis of the DLS data collected is performed as previously, in respective pairs of linear and hyperbranched quaterpolymer groups according to polymer composition, and any differentiation between them is discussed in the following. As far as the linear quaterpolymers are concerned, it seems that the formation of multichain aggregates is favored at both acidic and basic pH values. Due to their linear molecular structure, it is expected that the polymeric chains assemble into nanostructures, where the hydrophobic component is enclosed in the interior, and the pH-responsive acidic/basic units are dispersed on the outer part of the nanostructure in accordance with the respective solution pH. It is noteworthy that in both compositions, the scattered light intensity and the hydrodynamic radius do not change significantly, indicating that the overall particle mass and size remain the same. However, there is a noteworthy shift in the relative intensities of the particle populations at different pHs. Concerning acidic conditions (pH = 3), a portion of single-chain nanoparticles have been transformed into multichain ones, denoting phenomena such as swelling due to a combination of the neutralization of the carboxylate groups and the protonation of the amino groups. At basic pH (pH = 10), a similar shift is observed, but it is more pronounced for the LT2 polymer, where most single-chain and/or dimer/trimer particles have been converted to multichain aggregates. This species may consist of aggregates induced from the aggregation of polymer chains due to deprotonation of DMAEMA segments. It should be noted that zeta potential values follow the protonation/deprotonation equilibrium of the amphiphilic polyampholyte chains, showing positive values at acidic conditions and negative ones at basic conditions (Table 1).

As it was observed at neutral pH, macromolecular architecture has an important role in the assembly of these copolymers in aqueous solutions. Its effect is also important for acidic and basic pH values, and differentiations are observed for hyperbranched quaterpolymer in comparison with their linear analogues. In the case where the hydrophobic BzMA is at low proportion, monomodal distributions are maintained but become broader at both extreme pH values, indicating the existence of potentially larger polydisperse nanoparticles. The shift becomes more evident for HT2 solutions, where the induced swelling at pH = 3 results in broader distributions for both small and large nanoparticle populations. The surface charges between linear and hyperbranched copolymers obtained from ELS also show changes in the structure of the systems. At acidic conditions, a great deviation between LTs and HTs is observed, with the greater positive charge belonging to the hyperbranched quaterpolymers, assuming that DMAEMA units are more exposed to the solvent than in the case of the linear analogues.

### 3.4. Ionic Strength Effects

**Figure 6:**
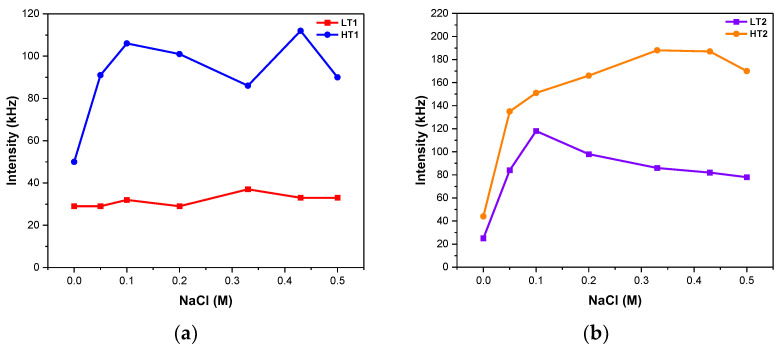
Scattered intensity from quaterpolymer solutions LT1/HT1 (**a**) and LT2/HT2 (**b**) as a function of ionic strength.

**Figure 7:**
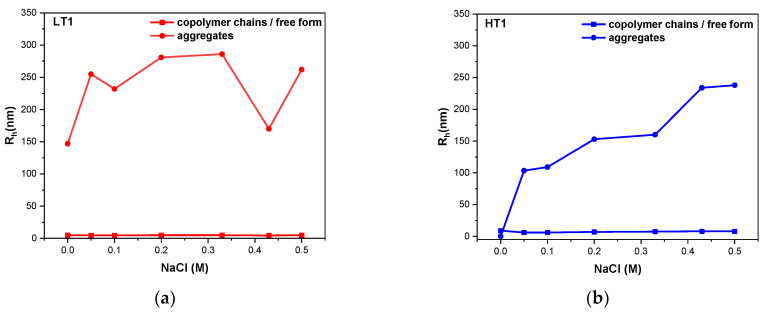

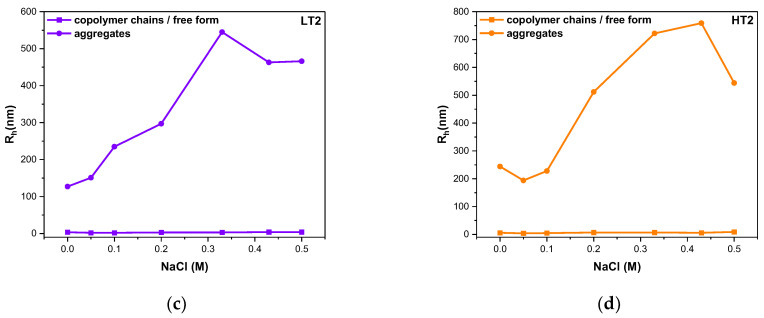
Hydrodynamic radius of quaterpolymers LT1 (**a**), HT1 (**b**), LT2 (**c**) and HT2 (**d**) as a function of ionic strength.

The effect of ionic strength on quaterpolymer solutions is investigated in order to examine potential alterations concerning the structure and conformation of the induced polymeric assemblies under specific salt conditions, as the addition of NaCl may affect the electrostatic interactions in the systems. The corresponding measurements were performed by utilizing the dynamic light-scattering technique and examining the change in scattered intensity (Figure 6) and hydrodynamic radius (Figure 7) as a function of six NaCl concentrations for each copolymer solution, at neutral pH.

Scattered intensity plots reveal possible alterations concerning the mass of the polymeric nanoparticles as the salt concentration increases. Intensity seems to increase more significantly for the hyperbranched quaterpolymers than for their linear analogues. This result comes in accordance with two factors that were elaborated in previous sections. The hyperbranched architecture together with the higher BzMA content imparts a drastic increase in the mass of HT2 nanoparticles as salt concentration increases and reaches 0.5 M. A respective increase in mass is also observed for HT1, but at a noticeably lower level. As for the linear analogues, the resulting nanoparticles of LT1 remain unaffected from the presence of salt, considering their mass, and those of LT2 show a lower increase.

The hydrodynamic radius plots also reveal some interesting trends concerning the effect of the hydrophobic content. In Figure 7, a slight increase in R_h_ values is depicted for the aggregates of quaterpolymers with lower BzMA content, but when BzMA content doubles, the increase in those values is huge and independent of the molecular architecture.

### 3.5. Interactions of Quaterpolymers’ FBS Components

**Figure 8:**
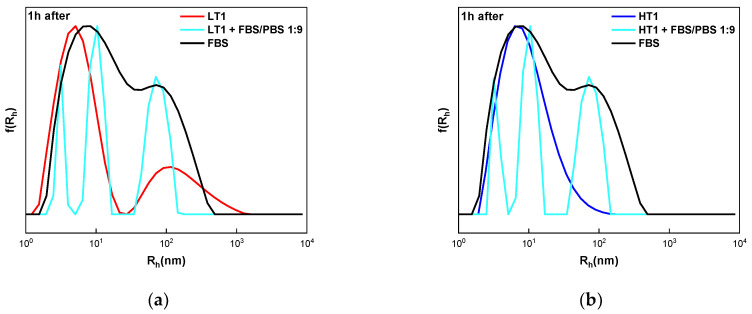

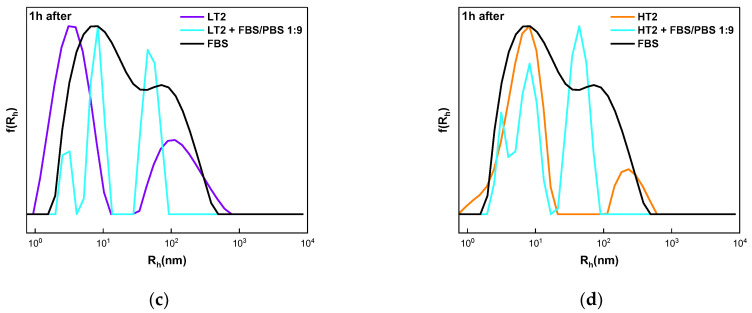
Size distributions from CONTIN analysis of DLS data for bare PBS, bare quaterpolymers LT1 (**a**), HT1 (**b**), LT2 (**c**), HT2 (**d**) and their mixtures in PBS solution after 1 h of mixing at 25 °C.

**Figure 9:**
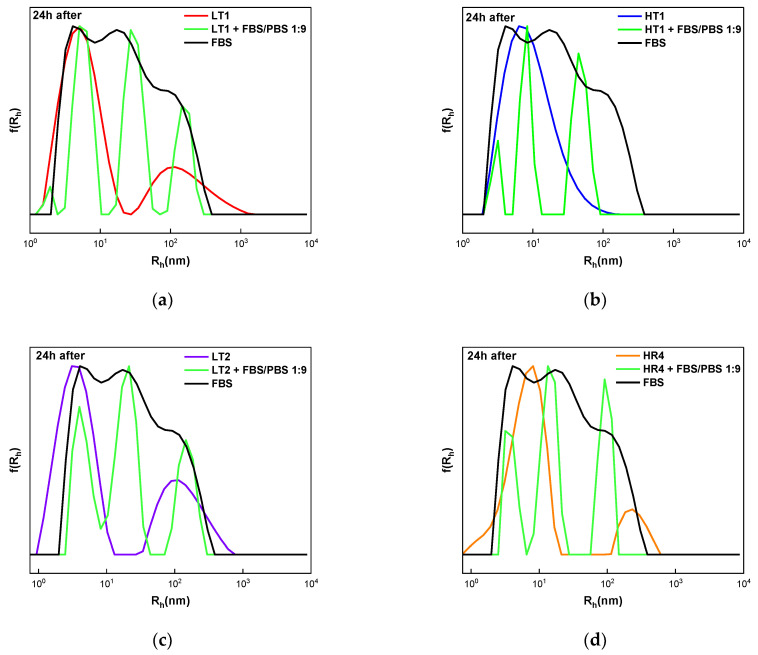
Size distributions from CONTIN analysis of DLS data for bare PBS, bare quaterpolymers LT1 (**a**), HT1 (**b**), LT2 (**c**), HT2 (**d**) and their mixture in PBS solution after 24 h of mixing at 25 °C.

Finally, the interactions between the quaterpolymer nano-assemblies synthesized with blood proteins were investigated under simulated physiological conditions. For this purpose, DLS measurements were conducted in FBS/PBS media, as described in the Materials and Methods section, to detect any possible alteration in their physicochemical properties. In Figure 8 and Figure 9, size distributions of the four quaterpolymers, pure FBS and their mixture are presented at two different timelines: an hour after and 24 h after their mixing, respectively. From the data in all eight plots, there are no indications that significant interactions between polymeric particles and blood proteins exist, since in all cases, nanometer-scale particles are observed (absence of microscale aggregates). This is a promising result, because it means that the quaterpolymers show remarkable stability in the simulated biological medium [28].

## 4. Conclusions

Four statistical linear and hyperbranched quaterpolymers of the types P(MAA-co-DMAEMA-co-OEGMA-co-BzMA) and H-P(MAA-co-DMAEMA-co-OEGMA-co-BzMA) were synthesized via RAFT polymerization. The polymers are complex systems showing amphiphilicity, polyampholyte character and aggregation properties in aqueous solutions, as the performed physicochemical characterization by light scattering and fluorescence spectroscopy indicate. It is interesting that a large proportion of the copolymers exist as single-chain particles in water. Their aggregation is enhanced in solution with higher ionic strengths (salting-out effect). Branching also promotes different aggregation behavior compared to the linear analogues, presumably because of the higher segment density and molecular topology of branched quaterpolymer chains. All these properties are reminiscent of the behavior of proteins. Quaterpolymers can be regarded as primitive models of natural proteins, and their aggregates may be useful in biomedical applications as multifunctional nanocarriers. 

## Figures and Tables

**Table 1:** DLS results for the four quaterpolymers in three different pHs.

Quaterpolymer	pH	I_90_° (kHz)	PDI	R_h_ (nm)	ζ-Potential (mV)
LT1	3	28.5	0.5	4 (44%)/95 (56%)	+16.2
	7	29	0.5	5 (75%)/147 (25%)	−11
	10	30	0.59	2 (21%)/135 (68%)	−51.5
HT1	3	40	0.55	18	+36.5
	7	50	0.43	9	−5
	10	55	0.5	51	−55
LT2	3	23.5	0.52	3 (44%)/133 (56%)	+30
	7	25	0.52	4 (68%)/127 (32%)	−6
	10	34	0.56	3 (20%)/99 (76%)	−48
HT2	3	38	0.54	6 (68%)/95 (32%)	+40
	7	44	0.46	6 (85%)/244 (15%)	−7
	10	38.5	0.52	19	−51

## Data Availability

Data are available upon request.

## Supplementary Materials

The following supporting information can be downloaded at https://www.mdpi.com/article/10.3390/polym16101321/s1: Figure S1: ^1^H-NMR spectrum of (a) LT2 (red) and (b) HT2 (green); Figure S2: Integrations of peaks “a” and “e” of linear quaterpolymers (a) LT1 and (b) LT2; Figure S3: Integrations of peaks “a” and “e” of hyperbranched quaterpolymers (a) HT1 and (b) HT2; Figure S4: ATR-FTIR spectra of (a) LT1/LT2 and (b) HT1/HT2; Figure S5: I_1_/I_3_ ratio as a function of the logarithm of polymer concentration for (a) LT1, (b) HT1, (c) LT2 and (d)HT2 at three different pHs; Figure S6: I_1_/I_3_ ratio of the highest quaterpolymer concentration as a function of pH for (a) LT1/HT1 and (b) LT2/HT2; Figure S7: Intensity-weighted size distribution and its respective correlation function for (a) LT1, (b) HT1, (c) LT2 and (d) HT2 at pH = 7.

## References

[1] Moad G., Rizzardo E., Thang S. (2008). Radical addition-fragmentation chemistry in polymer synthesis. Polymer.

[2] Nguyen T.D., Qiao B., Olvera de la Cruz M. (2018). Efficient encapsulation of proteins with random copolymers. Proc. Natl. Acad. Sci. USA.

[3] Sun Q., Yang Z., Qi X. (2023). Design and Application of Hybrid Polymer-Protein Systems in Cancer Therapy. Polymers.

[4] Hilburg S.L., Ruan Z., Xu T., Alexander-Katz A. (2020). Behavior of Protein-Inspired Synthetic Random Heteropolymers. Macromolecules.

[5] Jiang T., Hall A., Eres M., Hemmatian Z., Qiao B., Zhou Y., Ruan Z., Couse A.D., Heller W.T., Huang H. (2020). Single-chain heteropolymers transport protons selectively and rapidly. Nature.

[6] Sato K., Muraoka T., Kinbara K. (2021). Supramolecular Transmembrane Ion Channels Formed by Multiblock Amphiphiles. Acc. Chem. Res..

[7] Jayapurna I., Ruan Z., Eres M., Jalagam P., Jenkins S., Xu T. (2023). Sequence Design of Random Heteropolymers as Protein Mimics. Biomacromolecules.

[8] Panganiban B., Qiao B., Jiang T., DelRe C., Obadia M.M., Nguyen T.D., Smith A.A.A., Hall A., Sit I., Crosby M.G. (2018). Random heteropolymers preserve protein function in foreign environments. Science.

[9] Han Z., Hilburg S.L., Alexander-Katz A. (2022). Forced Unfolding of Protein-Inspired Single-Chain Random Heteropolymers. Macromolecules.

[10] Li L., Raghupathi K., Song C., Prasad P., Thayumanavan S. (2014). Self-assembly of random copolymers. Chem. Commun..

[11] Jiang W., Zhou Y., Yan D. (2015). Hyperbranched polymer vesicles: From self-assembly, characterization, mechanisms, and properties to applications. Chem. Soc. Rev..

[12] Soultan A.H., Verheyen T., Smet M., De Borggraeve W.M., Patterson J. (2018). Synthesis and peptide functionalization of hyperbranched poly(arylene oxindole) towards versatile biomaterials. Polym. Chem..

[13] Chakraborty A.K. (2001). Disordered heteropolymers: Models for biomimetic polymers and polymers with frustrating quenched disorder. Phys. Rep..

[14] Pande V.S., Grosberg A.Y., Tanaka T. (2000). Heteropolymer freezing and design: Towards physical models of protein folding. Rev. Mod. Phys..

[15] Mann S.K., Dufour A., Glass J.J., De Rose R., Kent S.J., Such G.K., Johnston A.P.R. (2016). Tuning the properties of pH responsive nanoparticles to control cellular interactions in vitro and ex vivo. Polym. Chem..

[16] Lutz J.-F. (2008). Polymerization of oligo(ethylene glycol) (meth)acrylates: Toward new generations of smart biocompatible materials. J. Polym. Sci. Part A Polym. Chem..

[17] Ozer I., Tomak A., Zareie H.M., Baran Y., Bulmus V. (2017). Effect of Molecular Architecture on Cell Interactions and Stealth Properties of PEG. Biomacromolecules.

[18] Bulmus V., Woodward M., Lin L., Murthy N., Stayton P., Hoffman A. (2003). A new pH-responsive and glutathione-reactive, endosomal membrane-disruptive polymeric carrier for intracellular delivery of biomolecular drugs. J. Control. Release.

[19] El-Sayed M.E.H., Hoffman A.S., Stayton P.S. (2005). Rational design of composition and activity correlations for pH-sensitive and glutathione-reactive polymer therapeutics. J. Control. Release.

[20] Keddie D.J., Moad G., Rizzardo E., Thang S.H. (2012). RAFT Agent Design and Synthesis. Macromolecules.

[21] Wilhelm M., Zhao C.L., Wang Y., Xu R., Winnik M.A., Mura J.L., Riess G., Croucher M.D. (1991). Poly(styrene-ethylene oxide) block copolymer micelle formation in water: A fluorescence probe study. Macromolecules.

[22] Xiao L., Liu C., Zhu J., Pochan D.J., Jia X. (2010). Hybrid, elastomeric hydrogels crosslinked by multifunctional block copolymer micelles. Soft Matter.

[23] Discher D.E., Eisenberg A. (2002). Polymer Vesicles. Science.

[24] Sengupta S., Das T., Ghorai U.K., Bandyopadhyay A. (2017). Copolymers from methyl methacrylate and butyl acrylate with hyperbranched architecture. J. Appl. Polym. Sci..

[25] Selianitis D., Pispas S. (2021). Multi-responsive poly(oligo(ethylene glycol)methyl methacrylate)-co-poly(2-(diisopropylamino)ethyl methacrylate) hyperbranched copolymers via reversible addition fragmentation chain transfer polymerization. Polym. Chem..

[26] Luo S., Han M., Cao Y., Ling C., Zhang Y. (2011). Temperature- and pH-responsive unimolecular micelles with a hydrophobic hyperbranched core. Colloid Polym. Sci..

[27] Balafouti A., Pispas S. (2023). Hyperbranched Copolymers of Methacrylic Acid and Lauryl Methacrylate H-P(MAA-co-LMA): Synthetic Aspects and Interactions with Biorelevant Compounds. Pharmaceutics.

[28] Wang X., Sun X., Jiang G., Wang R., Hu R., Xi X., Zhou Y., Wang S., Wang T. (2013). Synthesis of biomimetic hyperbranched zwitterionic polymers as targeting drug delivery carriers. J. Appl. Polym. Sci..

