# Supplementary material for: Four-Component Statistical Copolymers by RAFT Polymerization
Source: Polymers (Basel). 2024 May 8;16(10):1321. doi: 10.3390/polym16101321 (PMC11125712; doi:10.3390/polym16101321)
Supplement: Supplementary file 1 [file polymers-16-01321-s001.zip › polymers-2964099-supplementary.pdf]

LT2

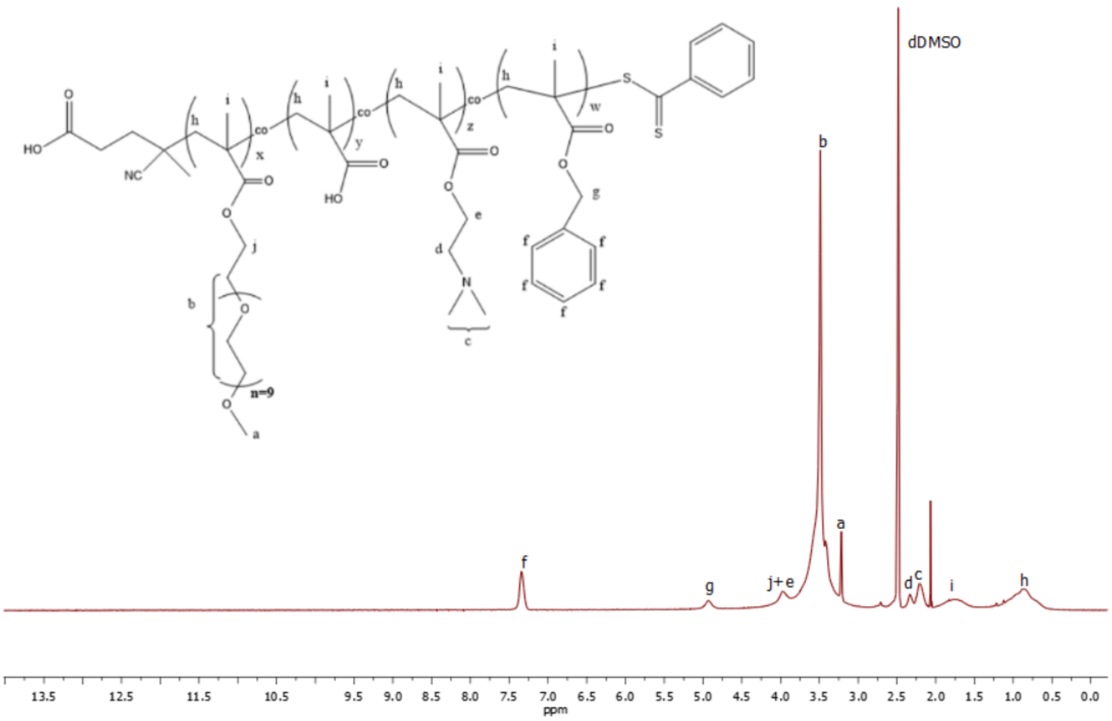
dDMSO
b
a
f
g
j+e
d
c
i
h
HO
NC
HO
co
x
y
z
w
n=9
S
13.5
12.5
11.5
10.5
9.5
9.0
8.5
8.0
7.5
7.0
6.5
6.0
5.5
5.0
4.5
4.0
3.5
3.0
2.5
2.0
1.5
1.0
0.5
0.0
ppm

(a)

HT2

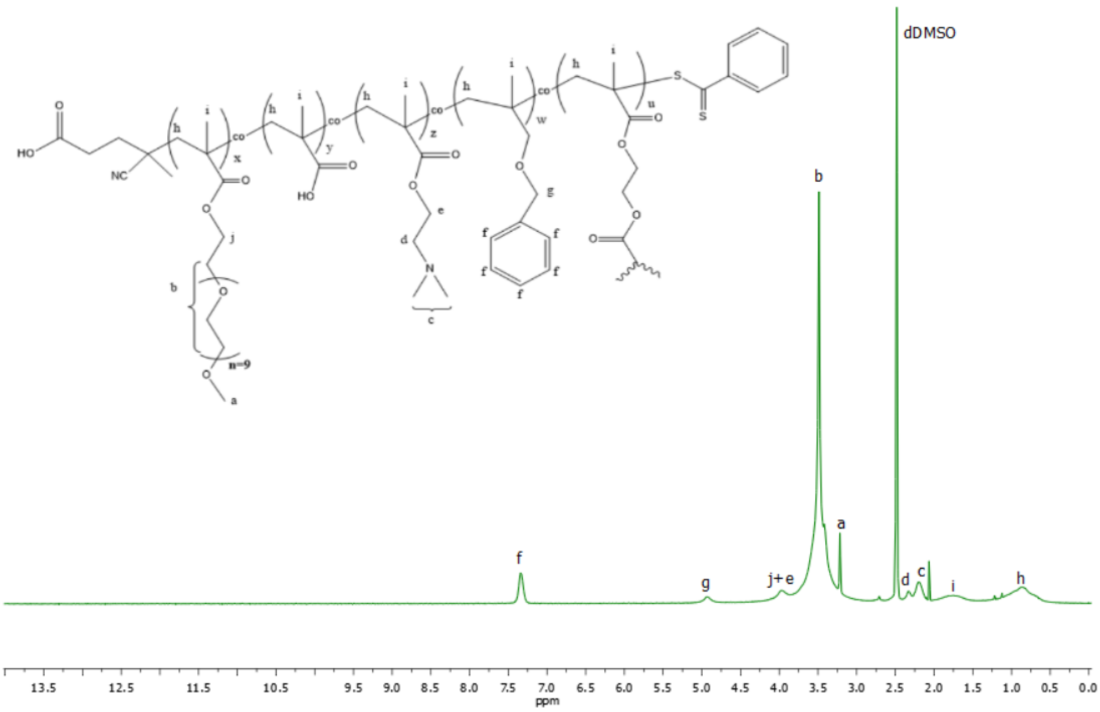

(b)

Figure S1. ^{1}H-NMR spectrum of (a) LT2 (red) and (b) HT2 (green).

LT1

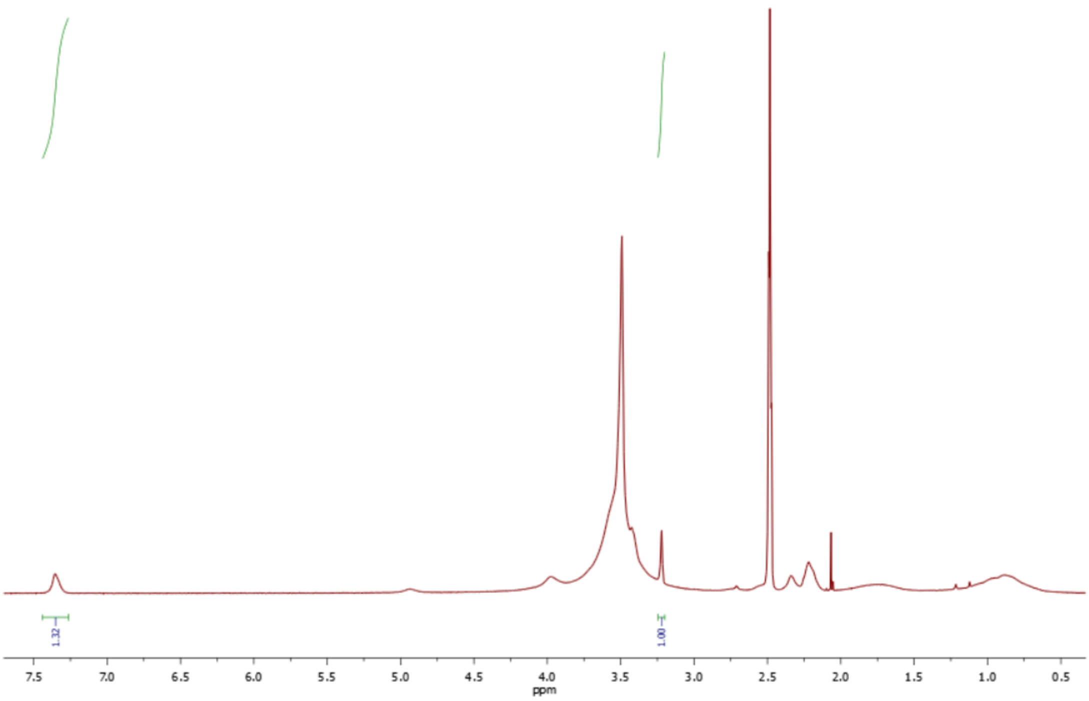

(a)

LT2

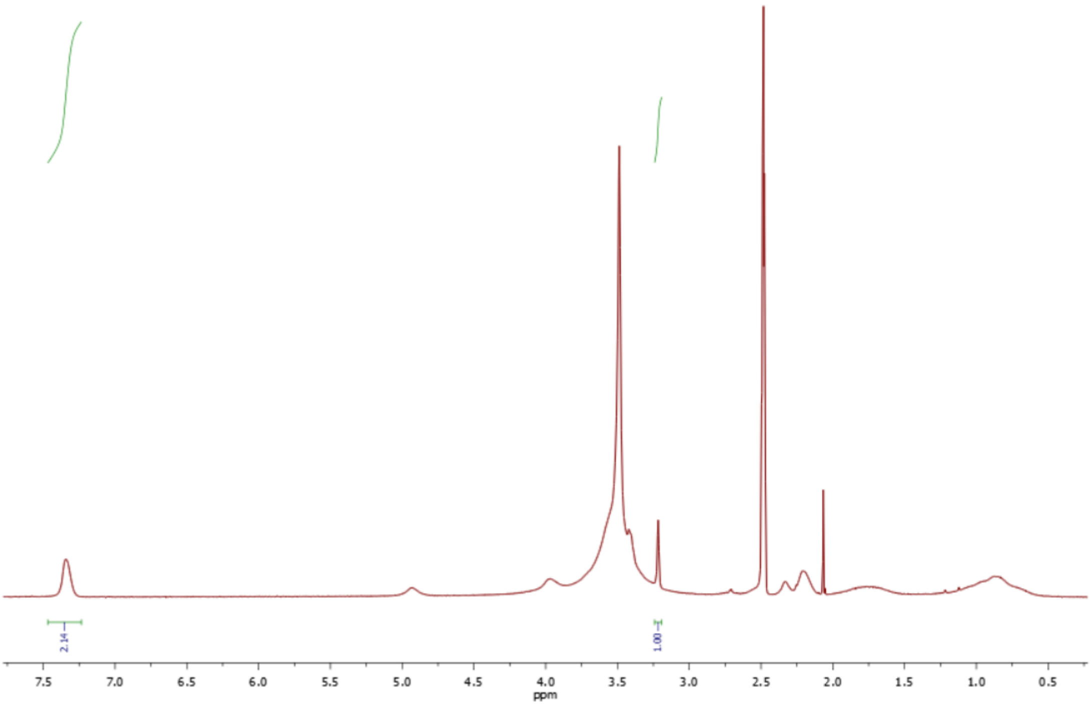

(b)

Figure S2. Integrations of peaks "a" and "f" of linear quaterpolymers (a) LT1 and (b) LT2.

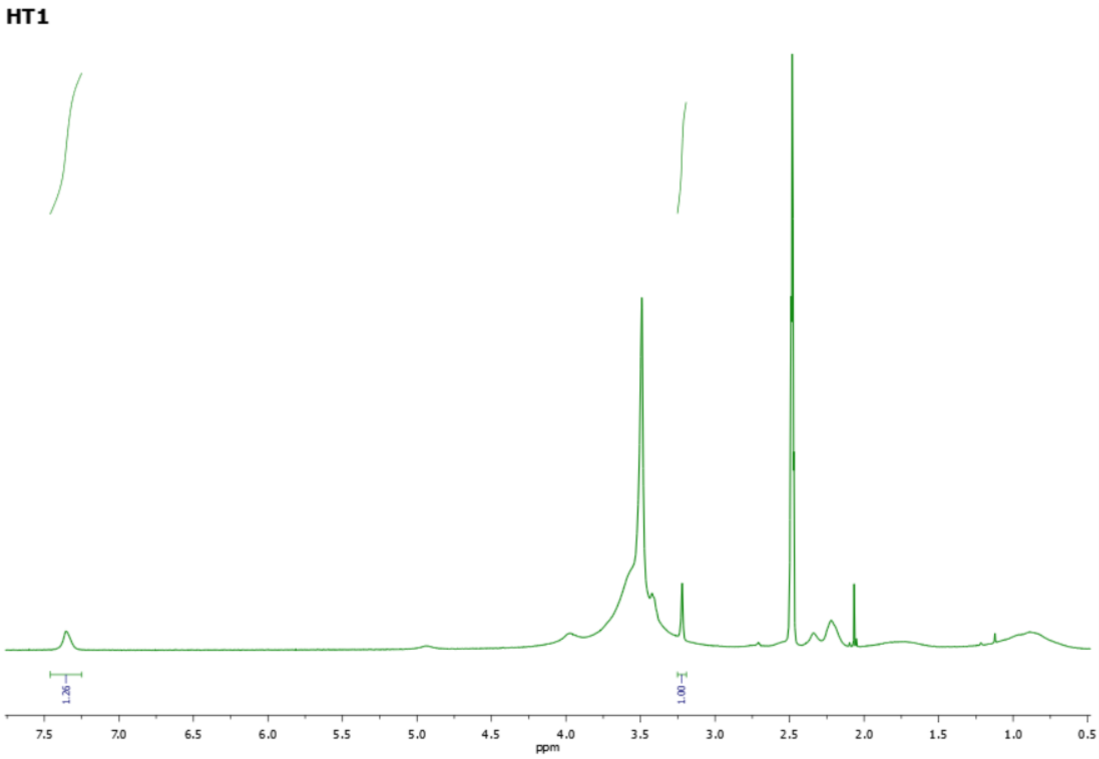

(a)

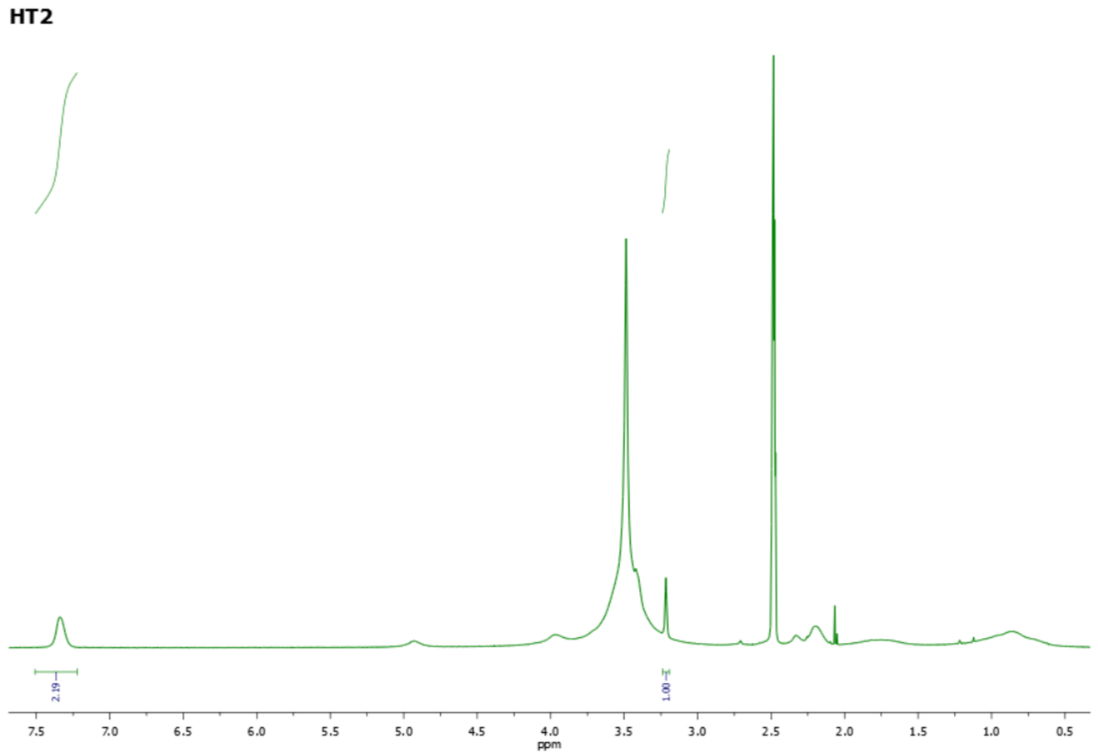

(b)

Figure S3. Integrations of peaks "a" and "f" of hyperbranched quaterpolymers (a) HT1 and (b) HT2.

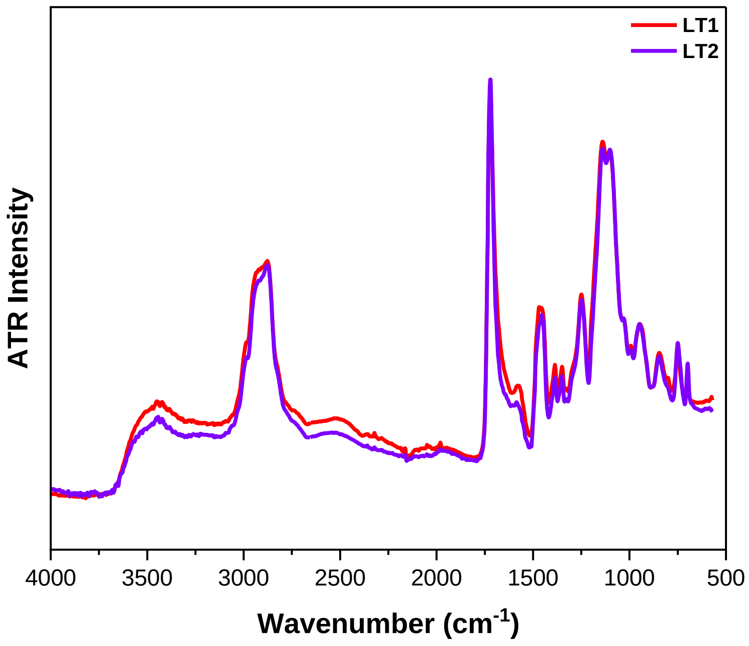

(a)

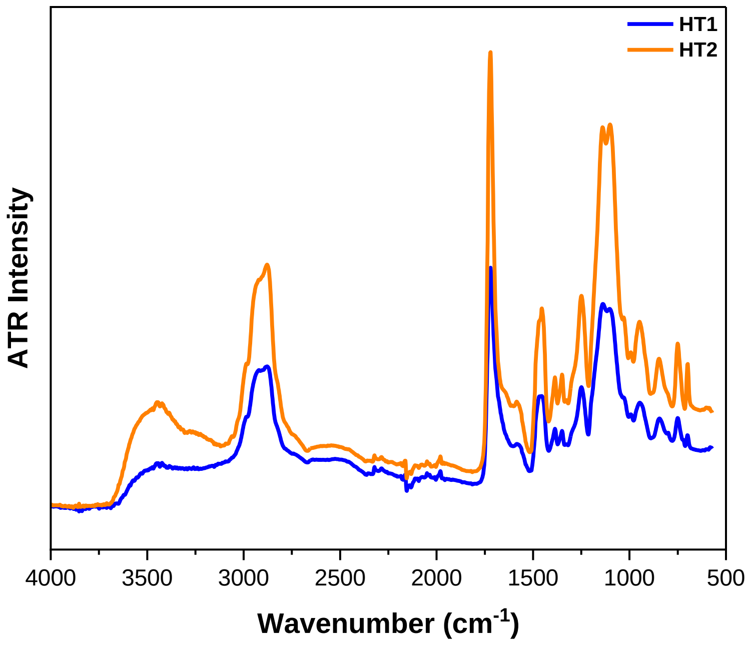

(b)

Figure S4. ATR-FTIR spectra of (a) LT1/LT2 and (b) HT1/HT2.

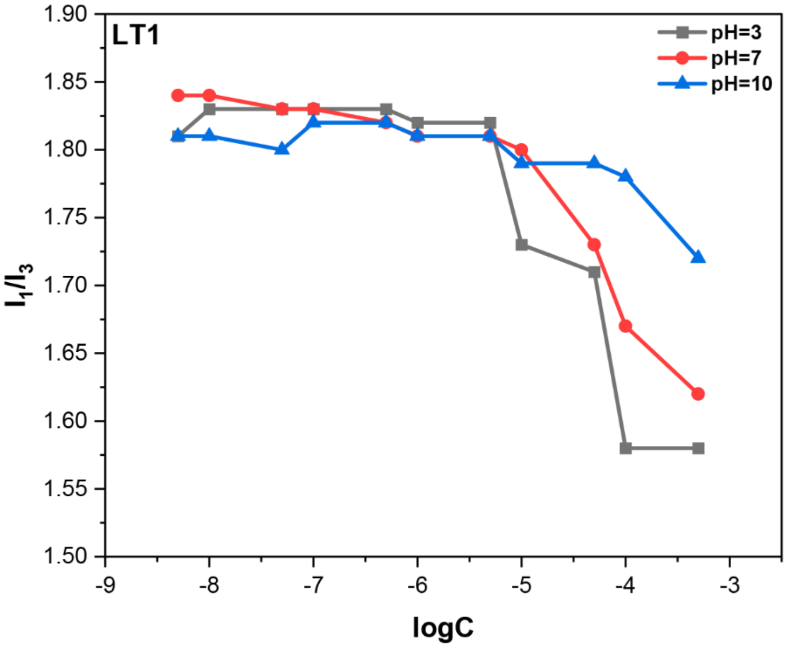
LT1
pH=3
pH=7
pH=10
1.90
1.85
1.80
1.75
1.70
1.65
1.60
1.55
1.50
I_1/I_3
-9
-8
-7
-6
-5
-4
-3
logC

(a)

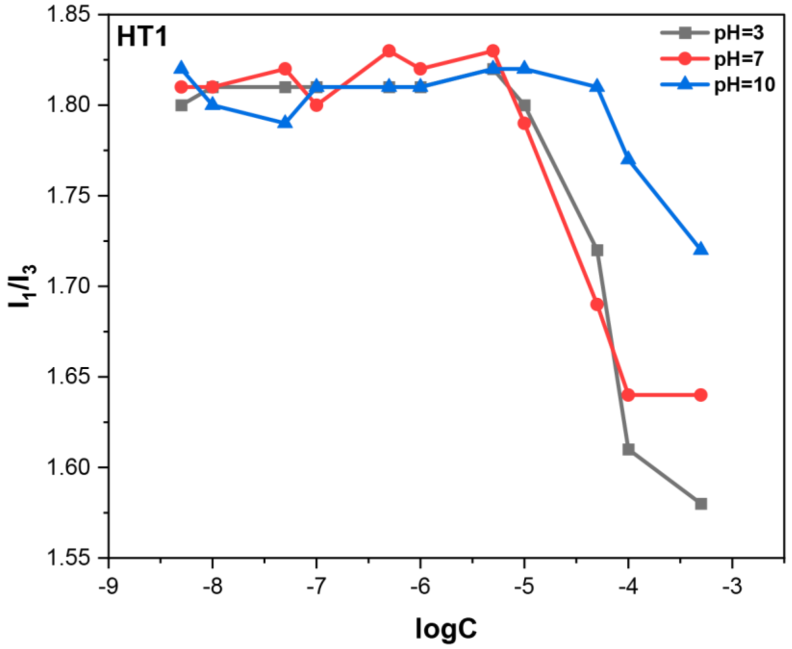
HT1
pH=3
pH=7
pH=10
1.85
1.80
1.75
1.70
1.65
1.60
1.55
I_1/I_3
-9
-8
-7
-6
-5
-4
-3
logC

(b)

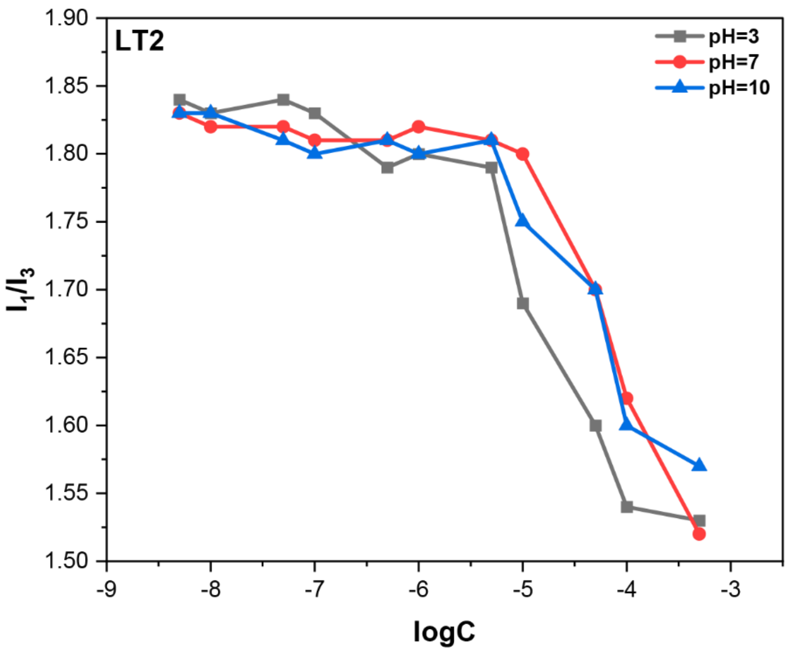

(c)

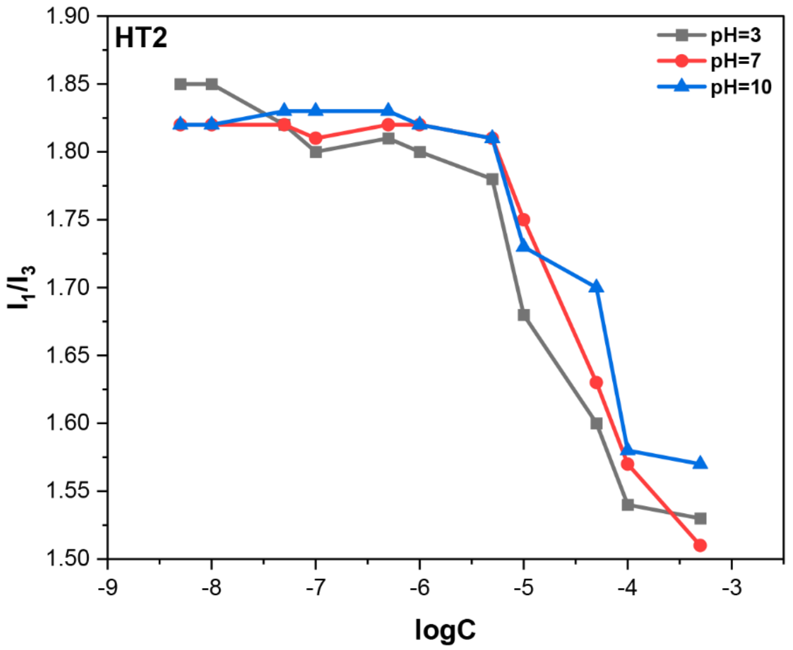

(d)

Figure S5. I_1/I_3 ratio as a function of the logarithm of (a) LT1, (b) HT1, (c) LT2 and (d) HT2 concentration at three different pHs.

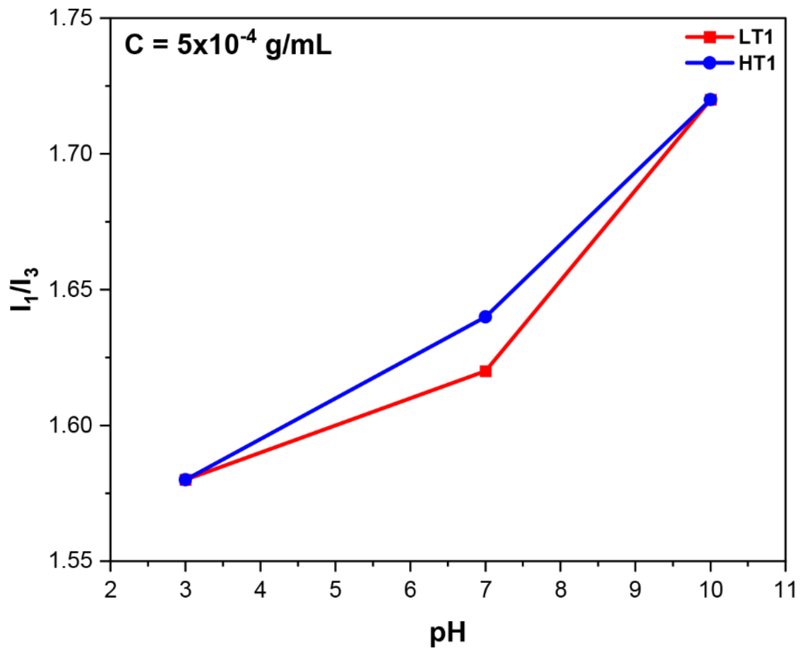

(a)

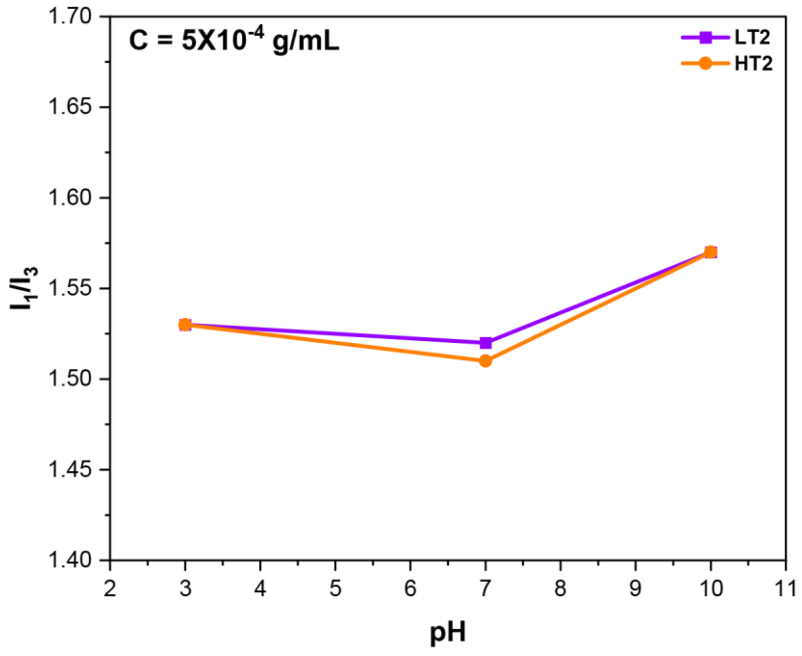

(b)

Figure S6. I_1/I_3 ratio of the maximum quaterpolymer concentration investigated (C = $5x10^{-4}$ g/mL) as a function of pH for (a) LT1 / HT1 and (b) LT2 / HT2 polymers.

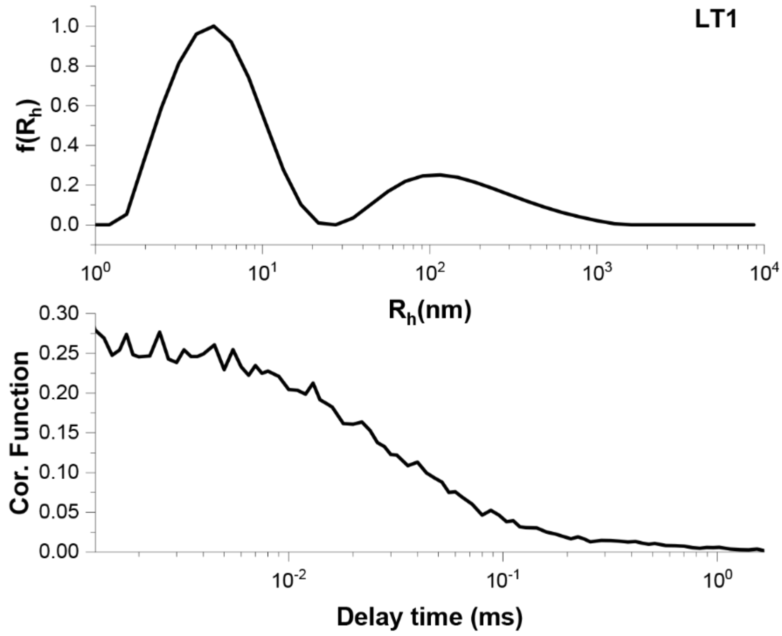
LT1
f(Rh)
Rh(nm)
Cor. Function
Delay time (ms)

(a)

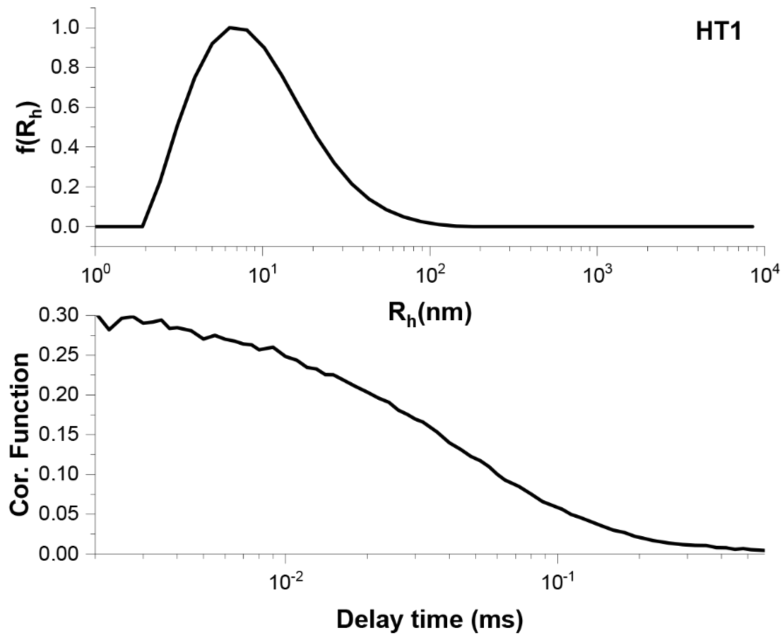
HT1
f(Rh)
Rh(nm)
Cor. Function
Delay time (ms)

(b)

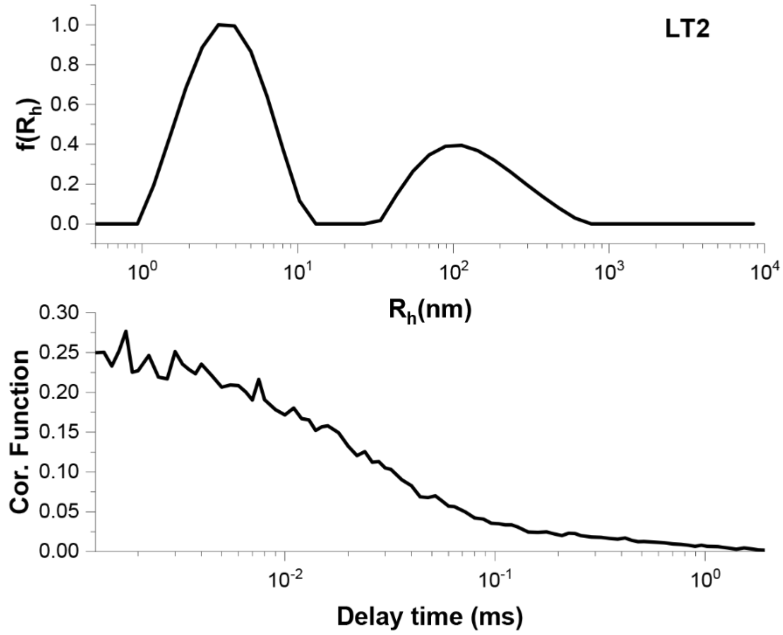

(c)

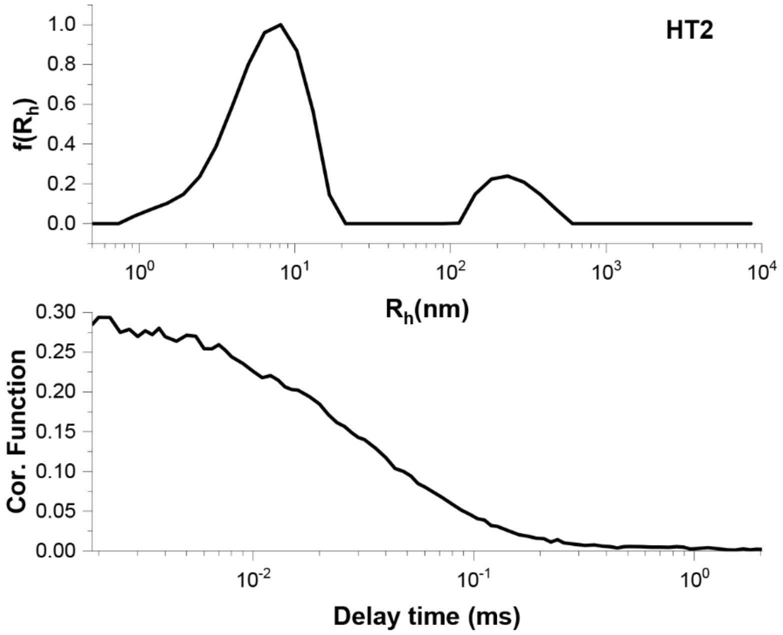

(d)

Figure S7. Intensity weighted size distribution and its respective correlation function for (a) LT1, (b) HT1, (c) LT2 and (d) HT2 at pH=7.
